# Determination of Transverse Shear Stiffness of Sandwich Panels with a Corrugated Core by Numerical Homogenization

**DOI:** 10.3390/ma14081976

**Published:** 2021-04-15

**Authors:** Tomasz Garbowski, Tomasz Gajewski

**Affiliations:** 1 Department of Biosystems Engineering, Poznan University of Life Sciences, Wojska Polskiego 50, 60-627 Poznan, Poland; tomasz.garbowski@up.poznan.pl; 2 Institute of Structural Analysis, Poznan University of Technology, Piotrowo 5, 60-965 Poznan, Poland

**Keywords:** corrugated board, numerical homogenization, strain energy equivalence, finite element method, plate stiffness properties, shell structures, transverse shear

## Abstract

Knowing the material properties of individual layers of the corrugated plate structures and the geometry of its cross-section, the effective material parameters of the equivalent plate can be calculated. This can be problematic, especially if the transverse shear stiffness is also necessary for the correct description of the equivalent plate performance. In this work, the method proposed by Biancolini is extended to include the possibility of determining, apart from the tensile and flexural stiffnesses, also the transverse shear stiffness of the homogenized corrugated board. The method is based on the strain energy equivalence between the full numerical 3D model of the corrugated board and its Reissner-Mindlin flat plate representation. Shell finite elements were used in this study to accurately reflect the geometry of the corrugated board. In the method presented here, the finite element method is only used to compose the initial global stiffness matrix, which is then condensed and directly used in the homogenization procedure. The stability of the proposed method was tested for different variants of the selected representative volume elements. The obtained results are consistent with other technique already presented in the literature.

## 1. Introduction

Corrugated cardboard is widely used as packaging and protective material in almost all industries. Whenever a product is displayed in shop windows, it is often packaged in colorful and branded corrugated cardboard packaging. This becomes a required standard all over the world. The packaging is not only to attract the eye of the customer, but is often the main protection for the product that is transported to warehouses or directly delivered to customers by courier companies. Along with the growth of e-commerce, the amount of packaging that goes to the market also grows. Fortunately, corrugated cardboard is a material that is not only environmentally friendly, but also easily recycled. These features largely contributed to the noticeable growth of the corrugated board packaging market in recent years. As a result of the growing awareness of producers and their customers, ecological products are gaining in popularity and therefore require more attention.

As long as the corrugated board is made of paper and the paper is made of cellulose fibers, which mainly come from trees, we must pay particular attention to the sustainable use of virgin and recycled fibers. The only way to achieve savings in the material used for the production of packaging is to focus the attention on the optimal selection of the composition of raw materials and a thorough strength analysis of corrugated board products. Currently, not only simple transport packages need to be optimized, but also more complex structures, e.g., SRP (shelf ready boxes) or displays. For typical box designs, it is sufficient to estimate the strength of a corrugated cardboard box on the basis of any analytical formula found in the literature; from the simplest and most popular [1] to the more complex [2,3,4,5,6,7].

McKee and coworkers developed the formula in which a compressive strength in cross direction of corrugated cardboard, its thickness and base dimension of the box is required to provide a simple estimation of the box strength. This approach is only valid for very simple flap boxes and can be used for regular shaped packages without perforation and holes. In the recent years many attempts were made to extend the applicability of simple analytical methods and to improve their accuracy. Allerby and coworkers modified constants and exponents in original McKee formulation which slightly improved its accuracy [2]. Schrampfer et al. extended the applicability of the McKee formula for wider range of boxes [8]. Batelka et al. included all box dimensions in their formula [3], while Urbanik et al. included also inelastic buckling phenomenon [4]. Recently, the numerical-analytical formula was proposed by Garbowski et al. to take into account also holes [6] and perforation [7] in the estimation of the box strength.

The strength of a slender box depends on the compressive strength of the corrugated board, but also on the critical load that its vertical walls must withstand. Therefore many research has been devoted to the phenomenon of corrugated board buckling [9,10,11,12,13]. Since corrugated board is a laminated material with a special fiber orientation, the buckling analysis requires advances models. Both the orthotropic nature of the material and its layered cross-section should be taken into account [14]. Therefore, the finite element method is the most appropriate method to calculate the critical load capacity of panels made of corrugated board. Especially in the case of complex shapes of such panels or in the presence of holes and perforations [6,7] where analytical formulas are difficult to apply.

In recent years, to assess the strength of corrugated cardboard structures, both hybrid methods [4,6,7,15] or purely numerical [16,17,18,19] have been increasingly used. A recent review can be found here [20]. Since corrugated cardboard boxes, fruit trays, displays and retail ready boxes are very often complex 3D structures loaded in various ways, the finite element method [21] is most often used for calculations of such structures. Corrugated board has a soft corrugated core, therefore the traditional Kirchhoff–Love plate theory is usually replaced with the Mindlin–Reissner shell theory, which also takes into account the transverse shear in the shell members. This require proper selection of the finite element (FE), which is of key importance for obtaining the correct results of numerical simulation. It is known that both triangular and quadrilateral shell FE suffer from a so-called shear locking. To overcome such limitations, many improvements to the traditional FE have been proposed in the literature, e.g., Bathe and Dvorkin [22,23], where auxiliary shear modes were applied. These modes was first used by MacNeal [24,25] and later extended by Done and Lamain [26] and Onate et al. [27]. This element has been successfully implemented and used in the work by Garbowski et al. [13], in which the authors prove that the mechanical behavior of this element in twisting tests is identical to the analytical predictions.

In case of structures made of corrugated boards very rarely the full multi-layered structure of the cross-section is modeled. Typically, a complex multi-layer cross-section is replaced with a single-layer model that has equivalent properties very similar to those of the full model. Such converting process is called homogenization. The homogenization of composite laminates has been the subject of interest of many researchers for several decades. One of the recent method that uses a strain energy was proposed in 2003 by Hohe [28] for homogenization of sandwich panels with hexagonal honeycomb core. The author uses a strain energy based procedure with assumed mechanical equivalence between a representative volume element (RVE) of a periodic plate and the simplified model, provided that the effective deformation in both models are equal in an average sense. Buanic et al. proposed a periodic homogenization method in which both an equivalent membrane, bending and shearing characteristics of periodic plates can be computed [29]. Biancolini obtained both membrane and bending properties for plates with corrugated core using the strain energy equivalence between the numerical model of RVE and single layered equivalent model [30]. The comparison of different approaches to homogenization of sandwich panels with corrugated boards can be found, e.g., in Garbowski and Jarmuszczak [31,32], and Marek and Garbowski [33]. The application of inverse analysis to homogenization of corrugated board was presented in the work of Garbowski and Marek [34].

An extension of the homogenization method proposed by Biancolini is presented here. The proposed generalization allows to take into account transverse shear in the process of homogenization of the corrugated cardboard. As already mentioned, transverse shear plays an important role in the mechanical behavior of the corrugated board, therefore many researchers have proposed different methods to calculate the effective transverse shear stiffness of the corrugated board [14,35,36,37,38]. This article presents the strain energy equivalence between RVE-base method of the full multi-layer corrugated cardboard FE model and the equivalent single-layer shell model. The proposed approach allows to calculate all properties of tensile, bending and transverse shear stiffnesses, which are extremely important if one would like to properly model the behavior of homogenized sandwich with corrugated cores. The method presented here has promising applications, not only to corrugated cardboards, but also for other types of sandwich or composite structures, including dynamic analysis, e.g., [39,40]. The results obtained by our method were compared with the results from the literature. A satisfactory agreement with the literature data was obtained.

## 2. Materials and Methods

The homogenization method proposed here is based on the equivalent of the deformation energy between a small part of a periodic multi-layer structure cut from corrugated cardboard and its simplified single-layer counterpart. Given the representative volume element (RVE) of the full detailed corrugated board model on the one hand and the simplified model on the other hand, the effective properties can be calculated, provided that the effective strains in both models are equal in an average sense. For the correct representation of the geometry of the cross-section a finite element models are used here.

**Figure 1:**
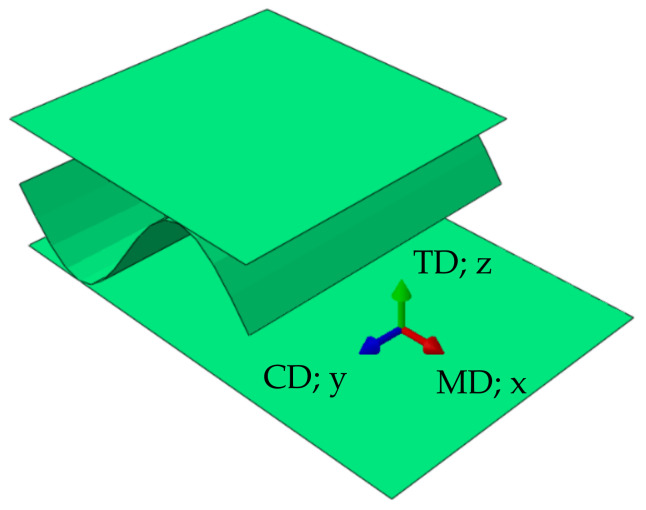
Material orientations.

Corrugated cardboard is a material made of several layers of paperboard. It consists of alternating flat and corrugated layers. The cellulose fibers in each of these layers are oriented along the waves, see Figure 1. This direction is called the machine direction (MD). The second, in plane direction, perpendicular to the fibers orientation, is called the cross direction (CD). The out of plane direction is the thickness direction.

**Figure 2:**
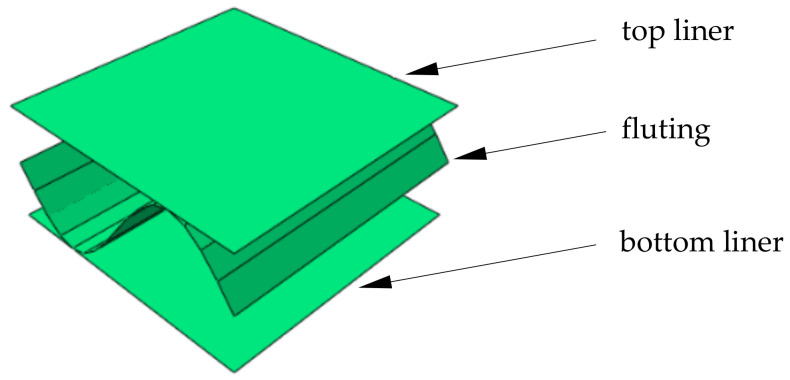
Representative volume element (RVE).

In order to compute all effective parameters of equivalent single-layered model, first the RVE need to be constructed. Here the single-wall corrugated cardboard is investigated therefore a selected RVE consists of singe period (see Figure 2) of the wavey layer. This selection was made to test the effect of the RVE type on the quality and stability of the calculated effective membrane, bending and transverse shear stiffnesses of the equivalent plate. The most problematic and least stable parameters identified by the homogenization method proposed here turned out to be both transverse shear stiffness in plane 13 (MD-TD) and 23 (CD-TD). Therefore, other RVE types and boundary conditions were also investigated in this study to check the robustness of the proposed approach.

**Figure 3:**
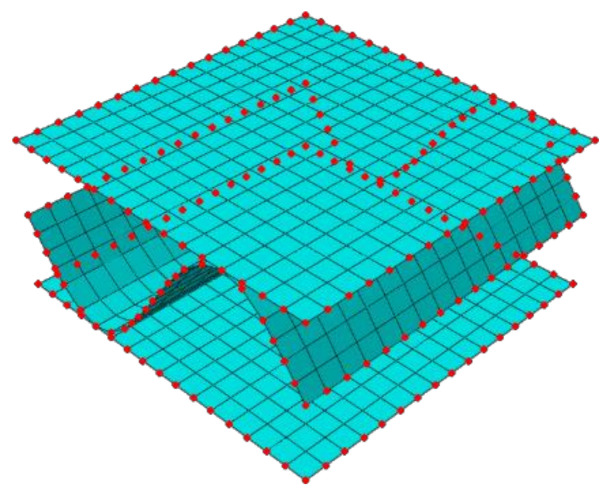
External (in red color) and internal nodes of RVE.

In the traditional displacement based linear formulation of finite element we have:

(1) $$K_{e}\;u_{e}=F_{e},$$

where $K_{e}$ is a statically condensed (through elimination of internal nodes) the global stiffness matrix of the RVE, $u_{e}$ is a displacement vector of the external nodes and $F_{e}$ is a vector of the nodal force applied to the external nodes. The FE mesh and external nodes are visualized in Figure 3.

The stiffness matrix condensed to external nodes can be computed by the following equation:

(2) $$K=K_{ee}-K_{ei}\;K_{ii}^{-1}K_{ie}$$

where overall stiffness matrix is partitioned into external (subscript e) and internal (subscript i) nodes into four submatrices in the following way:

(3) $$\left[\begin{array}{cc} K_{ee} & K_{ei}\\ K_{ie} & K_{ii} \end{array}\right]\left[\begin{array}{c} u_{e}\\ u_{i} \end{array}\right]=\left[\begin{array}{c} F_{e}\\ 0 \end{array}\right]$$

After static condensation (Equation (2)), the strain energy stored in the system is:

(4) $$E=\frac{1}{2}u_{e}^{T}\;F_{e}$$

The energetic equivalence between the FE model of the RVE and the simplified shell model can be established by a proper definition of the displacements and rotations in the external nodes. These general displacements at each boundary node are related to the generalized strains, which for membrane behavior reads:

(5) $$\left[\begin{array}{c} \varepsilon_{x}^{0}\\ \varepsilon_{y}^{0}\\ \gamma_{xy}^{0} \end{array}\right]=\left[\begin{array}{c} \partial u_{0}/\partial x\\ \partial v_{0}/\partial y\\ \partial u_{0}/\partial y+\partial v_{0}/\partial x \end{array}\right].$$

Displacements are related with rotations in the following way:

(6) $$\left\{\begin{array}{c} u\left(x,y,z\right)\\ v\left(x,y,z\right)\\ w\left(x,y,z\right) \end{array}\right\}=\left\{\begin{array}{c} -z\;\theta_{x}\left(x,y\right)\\ -z\;\theta_{y}\left(x,y\right)\\ w_{0}\left(x,y\right) \end{array}\right\},$$

while rotations according to Kirchhoff–Love assumption are considered as:

(7) $$\left\{\begin{array}{c} \theta_{x}\\ \theta_{y} \end{array}\right\}=\left\{\begin{array}{c} \partial w/\partial x\\ \partial w/\partial y \end{array}\right\}.$$

Since in Kirchhoff–Love plate theory the normal remains orthogonal to the middle plane after deformation, we have:

(8) $$\left\{\begin{array}{c} \partial u/\partial z\\ \partial v/\partial z \end{array}\right\}=\left\{\begin{array}{c} -\partial w/\partial x\\ -\partial w/\partial y \end{array}\right\}.$$

The normal strains can be than computed from Equations (6) and (7):

(9) $$\left[\begin{array}{c} \varepsilon_{x}\\ \varepsilon_{y}\\ \gamma_{xy} \end{array}\right]=\left[\begin{array}{c} \partial u/\partial x\\ \partial v/\partial y\\ \partial u/\partial y+\partial v/\partial x \end{array}\right]=-z\left[\begin{array}{c} \partial \theta_{x}/\partial x\\ \partial \theta_{y}/\partial y\\ \partial \theta_{x}/\partial y+\partial \theta_{y}/\partial x \end{array}\right]=-z\left[\begin{array}{c} \partial^{2}w/\partial x^{2}\\ \partial^{2}w/\partial y^{2}\\ 2\partial^{2}w/\partial x\partial y \end{array}\right],$$

while transverse shear can be computed from:

(10) $$\left[\begin{array}{c} \gamma_{xz}\\ \gamma_{yz} \end{array}\right]=\left[\begin{array}{c} \partial w/\partial x+\partial u/\partial z\\ \partial w/\partial y+\partial v/\partial z \end{array}\right]=\left[\begin{array}{c} 0\\ 0 \end{array}\right].$$

This assumption does not allow to calculate the transverse shear. Therefore, the Mindlin–Reissner theory should be applied, where the rotation is described by the formula:

(11) $$\left\{\begin{array}{c} \theta_{x}\\ \theta_{y} \end{array}\right\}=\left\{\begin{array}{c} \partial w/\partial x+\phi_{x}\\ \partial w/\partial y+\phi_{y} \end{array}\right\},$$

where the normal rotation is obtained as the sum of two rotations: (i) The corresponding slope of the middle plane of the plate and (ii) the additional rotation ϕ, which results from the lack of orthogonality of the normal to the middle plane after deformation. Consequently we have:

(12) $$\left\{\begin{array}{c} \partial u/\partial z\\ \partial v/\partial z \end{array}\right\}=\left\{\begin{array}{c} -\left(\partial w/\partial x+\phi_{x}\right)\\ -\left(\partial w/\partial y+\phi_{y}\right) \end{array}\right\}.$$

Now the transverse shear reads:

(13) $$\left[\begin{array}{c} \gamma_{xz}\\ \gamma_{yz} \end{array}\right]=\left[\begin{array}{c} \partial w/\partial x+\partial u/\partial z\\ \partial w/\partial y+\partial v/\partial z \end{array}\right]=\left[\begin{array}{c} \partial w/\partial x-\theta_{x}\\ \partial w/\partial y-\theta_{y} \end{array}\right]=\left[\begin{array}{c} -\phi_{x}\\ -\phi_{y} \end{array}\right],$$

while the curvatures are:

(14) $$\left[\begin{array}{c} \kappa_{x}\\ \kappa_{y}\\ \kappa_{xy} \end{array}\right]=-\left[\begin{array}{c} \partial \theta_{x}/\partial x\\ \partial \theta_{y}/\partial y\\ \partial \theta_{x}/\partial y+\partial \theta_{y}/\partial x \end{array}\right].$$

Using the Mindlin–Reissner theory the normal strains consists of membrane and bending behaviors as follow:

(15) $$\left[\begin{array}{c} \varepsilon_{x}\\ \varepsilon_{y}\\ \gamma_{xy} \end{array}\right]=\left[\begin{array}{c} \partial u/\partial x\\ \partial v/\partial y\\ \partial v/\partial x+\partial u/\partial y \end{array}\right]=\left[\begin{array}{c} \varepsilon_{x}^{0}\\ \varepsilon_{y}^{0}\\ \gamma_{xy}^{0} \end{array}\right]+z\left[\begin{array}{c} \kappa_{x}\\ \kappa_{y}\\ \kappa_{xy} \end{array}\right],$$

that permit to calculate (from Equations (13)–(15)) by integration the in plane displacement fields along *x*-axis as follows:

(16) $$u\left(x,y,z\right)=x\left(\varepsilon_{x}^{0}+z\kappa_{x}\right)+\frac{y}{2}\left(\gamma_{xy}^{0}+z\kappa_{xy}\right)-\frac{z}{2}\gamma_{xz},$$

and along *y*-axis as follows:

(17) $$v\left(x,y,z\right)=y\left(\varepsilon_{y}^{0}+z\kappa_{y}\right)+\frac{x}{2}\left(\gamma_{xy}^{0}+z\kappa_{xy}\right)-\frac{z}{2}\gamma_{yz},$$

while out of plane displacements are:

(18) $$w\left(x,y\right)=-\frac{x^{2}}{2}\kappa_{x}-\frac{xy}{2}\kappa_{xy}-\frac{y^{2}}{2}\kappa_{y}-\frac{x}{2}\gamma_{xz}-\frac{y}{2}\gamma_{yz}.$$

Recalling the definition of curvatures in Equation (14) and after a first integration of angular rotation with respect to *x*-axis, the following rotation with respect to *y*-axis is obtained:

(19) $$\theta_{x}\left(x,y\right)=\phi_{x}+\frac{\partial w}{\partial x}=-y\kappa_{y}-\frac{x}{2}\kappa_{xy},$$

while the rotation with respect to *x*-axis is:

(20) $$\theta_{y}\left(x,y\right)=x\kappa_{x}+\frac{y}{2}\kappa_{xy}.$$

The originally proposed by Biancolini [30] and here extended (by taking into account also both transverse shear) relationship between generalized constant strains and the position of the external nodes can be expressed by the following transform:

(21) $$u_{i}=A_{i}\;\epsilon_{i},$$

where for single node ($x_{i}=x$, $y_{i}=y$, $z_{i}=z$) we have:

(22) $${\left[\begin{array}{c} u_{x}\\ u_{y}\\ u_{z}\\ \theta_{x}\\ \theta_{y} \end{array}\right]}_{i}={\left[\begin{array}{cccccccc} x & 0 & y/2 & z/2 & 0 & xz & 0 & yz/2\\ 0 & y & x/2 & 0 & z/2 & 0 & yz & xz/2\\ 0 & 0 & 0 & x/2 & y/2 & -x^{2}/2 & -y^{2}/2 & -xy/2\\ 0 & 0 & 0 & 0 & 0 & 0 & -y & -x/2\\ 0 & 0 & 0 & 0 & 0 & x & 0 & y/2 \end{array}\right]}_{i}{\left[\begin{array}{c} \varepsilon_{x}\\ \varepsilon_{y}\\ \gamma_{xy}\\ \gamma_{xz}\\ \gamma_{yz}\\ \kappa_{x}\\ \kappa_{y}\\ \kappa_{xy} \end{array}\right]}_{i}.$$

Recalling the definition of the strain energy for the discrete model:

(23) $$E=\frac{1}{2}u_{e}^{T}\;K\;u_{e}=\frac{1}{2}\epsilon_{e}^{T}\;A_{e}^{T}\;K\;A_{e}\;\epsilon_{e},$$

and considering that for a shell subjected to bending, traction and transverse shear the internal energy is:

(24) $$E=\frac{1}{2}\epsilon_{e}^{T}\;A_{k}\;\epsilon_{e}\left\{area\right\},$$

overall stiffness matrix for the laminate could be easily extracted from the discrete matrix as:

(25) $$A_{k}=\frac{A_{e}^{T}\;K\;A_{e}}{area}.$$

## 3. Results

The numerical examples presented in the study are referring to the material and geometrical data used in the work of Biancolini [30]. In the Table 1, the material properties used in this paper for liners and fluting are shown, namely, $E_{1}$, $E_{2}$, $v_{12}$, $G_{12}$, $G_{13}$, and $G_{23}$, i.e., Young moduli in both directions, Poisson’s ratio and shear moduli, respectively. Also, the paper thicknesses, t, are shown in Table 1. The fluting period used here equals 8 mm. Apart Section 3.1, the axial spacing between internal and externa liners equals 3.51 mm. In Section 3.1, the axial spacing between liners itself was analyzed.

### 3.1. Stiffnesses Variation Due to Different Approach for Modelling Cross-Direction Section

**Figure 4:**
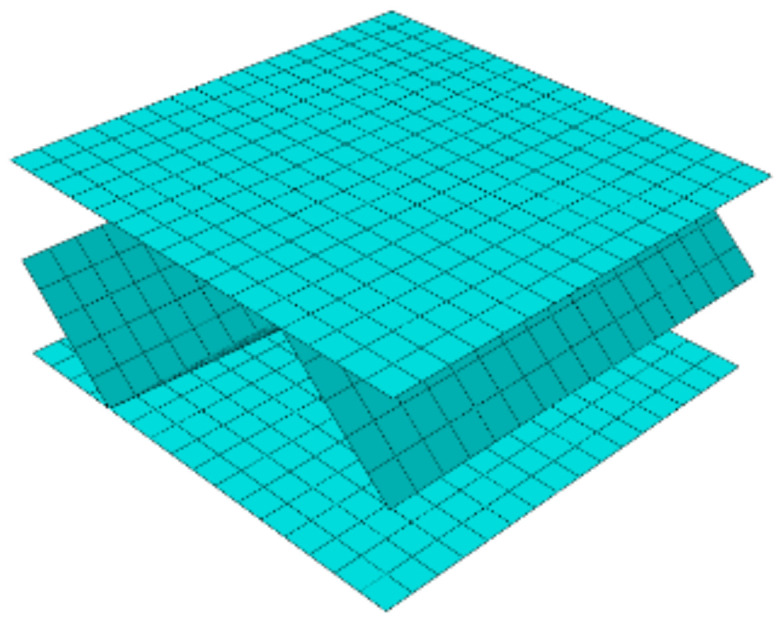
Representative shell elements of saw tooth geometry with quadrilateral mesh (single period).

**Figure 5:**
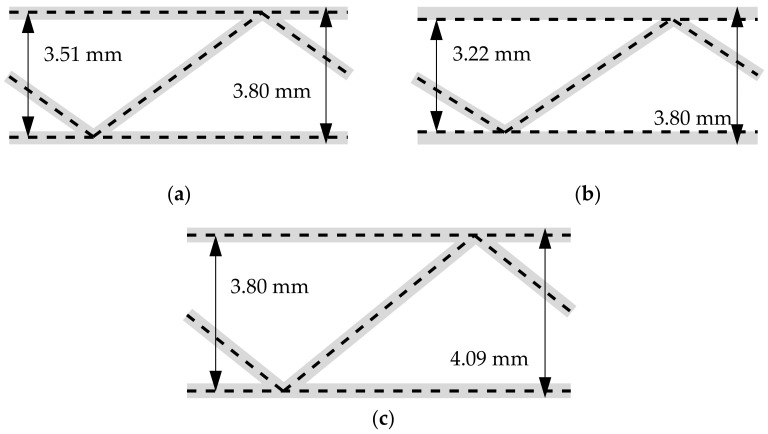
The different approach of modelling the cardboard cross-direction section of the saw tooth geometry: (**a**) 3.80 mm as the outer cardboard dimension, (**b**) 3.80 mm as the outer cardboard dimension with offset technique used and (**c**) 3.80 mm as axial spacing between liners.

In the first step of numerical part of the study, the examples presented by Biancolini [30] were used as reference and recreated. The saw tooth type geometry was considered here, see Figure 4. In the referred paper only the overall data regarding the geometry were explicitly given, there was a lack of detailed information about the modelling of the cross-section geometry. For instance, if the height of 3.8 mm used, was the overall outer thickness of the cardboard or the axial distance between the liners. Thus, in this study, we have utilized different approaches to model the cross-section geometry, see Figure 5, to verify which approach was used by the author. In Figure 5a, the axial spacing between shell liners equals 3.51 mm; the outer thickness equals 3.8 mm. In Figure 5b, the shells with offset technique were adopted; in this case the outer thickness was also 3.8 mm. In Figure 5c, the axial spacing between the shell liners equals 3.8 mm; the outer thickness equals 4.09 mm. In numerical examples of this section, the 4-node quadrilateral element with full integration scheme (labelled in Abaqus FEA as S4) was used.

Our computational results for saw tooth geometry are presented in Table 2. In the second column, the values according to [30] were demonstrated. In the third, fourth and fifth columns, the results computed using different geometry are presented, see Figure 5a–c, respectively and Materials and Methods section.

### 3.2. Stiffnesses Variation Due to Different Finite Element Type

In this section, the influence of using different element type in RVE on determination of $A_{k}$ stiffnesses was verified. Here, the sine geometry of fluting was used. In Table 3, the second column represents the results from the model with the 4-node quadrilateral element with full integration scheme (labelled in Abaqus FEA as S4). The third column represents the results from the model with the 4-node quadrilateral element with a reduced integration scheme (labelled in Abaqus FEA as S4R). The fourth column represent the results from the model with the 3-node triangular element (labelled in Abaqus FEA as S3). In the fifth column, the results for quadrilateral, bilinear deflection and rotations and linear transverse shear strain fields (QLLL) element was shown, embedded in in-house finite element method code [13]. In all cases, the number of nodes is the same, however, in the mesh with triangular element type the number of elements is almost twice bigger, see Table 3.

### 3.3. Stiffnesses Variation Due to Different Fluting Discretization

**Figure 6:**
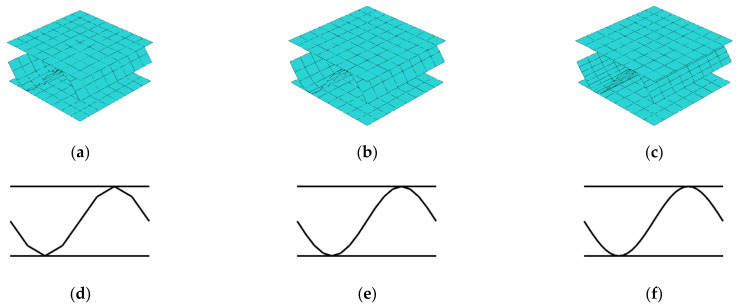
Different discretizations of cardboard fluting for unsymmetric RVE: (**a**) 8, (**b**) 16, and (**c**) 32 fluting segments; and corresponding cross-sections: (**d**) 8, (**e**) 16, and (**f**) 32 fluting segments.

Next, the fluting shape discretization was analyzed to derive, how the number of segments influence the determination of $A_{k}$ matrix. For this purpose different discretizations were considered, namely, 4, 8, 12, 16, 20, 24, 28, 32, 36, 40, 44, 48, 52, 56, 60, and 64 segments for a single fluting period. Two RVS were selected, the one with unsymmetric fluting (flute period starts from the middle), and the one with symmetric fluting (flute period starts from the liner). Three selected discretizations with 8, 16, and 32 segments on unsymmetric model are presented in the Figure 6. In the first row, the three-dimensional fluting cardboards are presented, in the second row the corresponding cross-sections are shown. In those numerical examples, the quadrilateral, bilinear deflection and rotations and linear transverse shear strain fields (QLLL) element was used.

**Figure 7:**
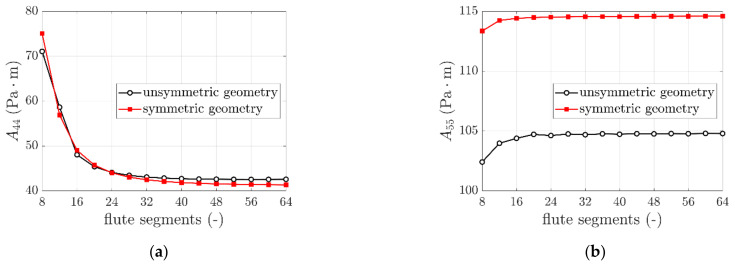
The variation of (**a**) $A_{44}$ and (**b**) $A_{55}$ due to different number of fluting segments used.

The $A_{k}$ stiffnesses obtained for those cases are presented in Table 4. In Figure 7, the results of $A_{44}$ and $A_{55}$ for all used flute segments (16 cases) are plotted separately.

### 3.4. Stiffnesses Variation Due to Different Numbers of Periods

**Figure 8:**
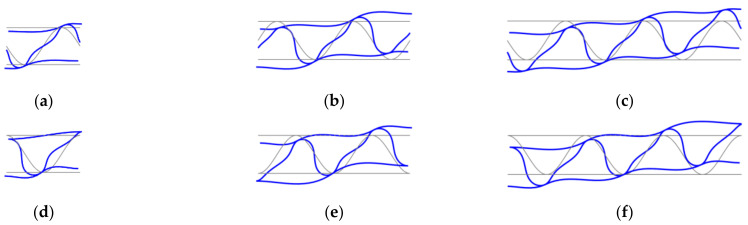
Deformation of RVE cross-section under transverse shear strains for different numbers of periods of corrugated cardboard for unsymmetric fluting cardboards: (**a**) 1, (**b**) 2, and (**c**) 3 periods; and symmetric fluting cardboards: (**d**) 1, (**e**) 2, and (**f**) 3 periods.

**Figure 9:**
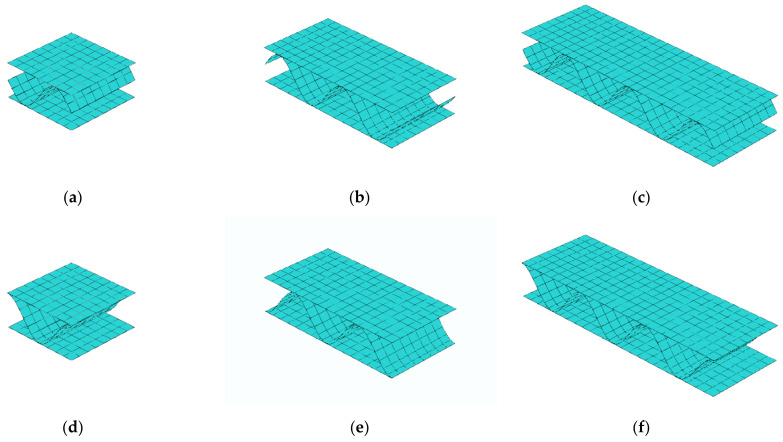
Different numbers of periods of corrugated cardboard for unsymmetric fluting cardboards: (**a**) 1, (**b**) 2, and (**c**) 3 periods; and symmetric fluting cardboards: (**d**) 1, (**e**) 2, and (**f**) 3 periods.

Because the application of general strains ($\gamma_{13}$) at RVE edges allows free deformation of liners and fluting (see Figure 8) therefore the influence of the number of periods of the internal layer on the calculated transversal shear stiffness $A_{44}$ was checked here. The different numbers of periods (namely 1, 2, or 3 periods) for corrugated cardboard with sine-shaped fluting was studied. Two geometries were analyzed, i.e., with the period starting from the middle of fluting–unsymmetric, see Figure 9a–c; and with the period starting from the liner–symmetric, see Figure 9d–f. In those numerical examples, the quadrilateral, bilinear deflection and rotations and linear transverse shear strain fields (QLLL) element was used. Note that in CD the length is conservatively assumed to be equal the period length, i.e., 8 mm. In Table 5, the second to fourth columns represent the results from the model with the unsymmetric periods—1, 2, or 3, respectively. The fifth to seventh columns represent the results from the symmetric periods—1, 2, or 3, respectively.

## 4. Discussion

### 4.1. Different Approach of Modelling Cross-Direction Section

Regarding results presented in Section 3.1 concerning modelling cross-direction section it should be noted, that the extended approach derived in this paper, in which $A_{44}$ and $A_{55}$ are computed from the RVE, does not influence the computed values of $A_{11}$, $A_{22},$ $A_{12},$ $A_{33},$ $D_{11}$, $D_{22}$, $D_{12}$, and $D_{33}$. Therefore, the data in the second column from Biancolini [30] may be directly compared with the third, fourth, and fifth columns. The stiffness in the second and third column are the closest to each other, thus, it may be concluded that this approach was used by author.

Notice that the inner geometry case (fourth column) is closer to the real-world geometry, but the offset technique used here is rarely available in finite element method software. Via comparing the third and the fourth columns, it may be concluded, that the inner geometry case does not give meaningful changes to the axial geometry case. Thus, the fluting simplification with the axial geometry case, without the use of the offset technique, is justified. On contrary, the outer geometry case meaningfully differs with other cases, especially in $D_{11}$, $D_{22}$, $D_{12},$ and $D_{33}$, in which distance between liners plays important role. In this case, corrugated cardboard thickness is 0.29 mm higher than in previous cases, cf. Figure 5c with Figure 5a,b.

### 4.2. Different Finite Element Type

Regarding results presented in Section 3.2 concerning different finite element type, while comparing results from using quadrilateral elements (second column) and results from using quadrilateral elements with reduced integration scheme (third column), it may be observed that all $A_{k}$ corresponding stiffnesses are very similar (difference less than 0.5%). There is no significant difference between the full quadrilateral and reduced quadrilateral element in $A_{44}$ and $A_{55}$.

While comparing the results from using quadrilateral elements (second column) and results from using triangular elements (fourth column), it may be observed that again $A_{k}$ corresponding stiffnesses are very close to each other (difference less than 0.5%). Here, there are some differences between full quadrilateral and triangular element in $A_{44}$ and $A_{55}$, 2.3% and 2.3%, respectively.

On the other hand, the differences obtained from QLLL and S4 elements are quite large, the most significant differences was in $A_{44}$ and $A_{55}$, i.e., about 27% and 46%, respectively. Since, this element approach was proved to be exceeding the S4/S4R/S3 elements, see [13], QLLL element was used in computations in Section 3.3 and Section 3.4.

### 4.3. Different Fluting Discretization

Regarding results presented in Section 3.3 concerning different fluting discretizations considered, while comparing unsymmetric and symmetric cases the results from using 32 segments (fourth column) with the results from using 16 and 8 segments, it may be observed that $A_{k}$ corresponding stiffnesses are similar. The difference less is than 1.7%. However, it should be noted that, as presented in Table 4 and Figure 7 there is a meaningful difference between the values of $A_{44}$ and $A_{55}$ considered for different segments number; it stabilizes with increasing number of fluting segments. As presented in Figure 7 an asymptote is reached for approximately 32 segments. The same effect is shown for both cases analyzed (unsymmetric and symmetric period).

### 4.4. Different Numbers of Periods

Regarding results presented in Section 3.4 concerning numbers of periods used, it may be noted that between unsymmetric period and symmetric period cases the differences in $A_{k}$ corresponding stiffnesses are negligible. The biggest differences are visible for $A_{44}$ and $A_{55}$, but they are still less than 5%, while for other stiffnesses they are less than 2%, which proves that the obtained results are independent of the RVE size.

## 5. Conclusions

In this research study, the homogenization technique for corrugated cardboard shell structures was considered, however it may be adopted for any periodic shell structure. The strain energy equivalence with condensation technique used to determine the stiffness properties of homogenized shell was extended here to determine not only the membrane and bending stiffnesses but also the transverse shear stiffnesses of any periodic shell structure. The techniques requires computing the FE global stiffness matrix of the full 3D FE shell structure and simple algebraic operations.

Based on this study several guidelines may be defined for robust determination of membrane, bending and transverse shear stiffnesses of corrugated cardboard. If one would like to acquire only membrane and bending stiffnesses the RVE selectin, in particular the fluting segments number or unsymmetric/symmetric geometry do not play any important role. But it should be noted that in order to determine proper values of transverse shear stiffnesses of the corrugated cardboard, at least 32 segments must be used for correct reconstruction of sine-shaped fluting. Furthermore, the selected number of periods in RVE is not affecting the obtained results, assuming the RVE dimension in CD length is constant. The presented here homogenization method together with practical guidelines can be successfully used to obtain stiffness properties of any corrugated shell structures.

## Figures and Tables

**Table 1:** Thicknesses and material properties of liners and fluting used in this study.

Layers	t (mm)	$E_{1}$ (MPa)	$E_{2}$ (MPa)	$\nu_{12}$ (-)	$G_{12}$ (MPa)	$G_{13}$ (MPa)	$G_{23}$ (MPa)
liners	0.29	3326	1694	0.34	859	429.5	429.5
fluting	0.30	2614	1532	0.32	724	362	362

**Table 2:** The stiffnesses of representative shell element computed for a different approach of modelling confronted with data from ref. [30] for saw tooth geometry.

Stiffness	Ref. [30]	Axial Geometry	Inner Geometry	Outer Geometry
$A_{11},\;\left(\mathrm{kPa}\cdot m\right)$	2158	2140	2154	2131
$A_{22},\;\left(\mathrm{kPa}\cdot m\right)$	1660	1665	1643	1687
$A_{12},\;\left(\mathrm{kPa}\cdot m\right)$	379.9	382.9	385.4	381.9
$A_{33},\;\left(\mathrm{kPa}\cdot m\right)$	677.6	662.5	668.4	656.8
$D_{11},\;\left(\mathrm{Pa}\cdot m^{3}\right)$	6.370	6.392	6.389	7.482
$D_{22},\;\left(\mathrm{Pa}\cdot m^{3}\right)$	3.824	3.859	3.740	4.549
$D_{12},\;\left(\mathrm{Pa}\cdot m^{3}\right)$	1.092	1.115	1.113	1.305
$D_{33},\;\left(\mathrm{Pa}\cdot m^{3}\right)$	1.655	1.656	1.639	1.937
$A_{44}$,$\left(\mathrm{Pa}\cdot m\right)$	-	202.4	179.4	218.5
$A_{55}$,$\;\left(\mathrm{Pa}\cdot m\right)$	-	99.0	89.0	112.4

**Table 3:** The stiffnesses of the representative shell element computed for different element type-sine geometry.

Stiffness	Quadrilateral Element (S4)	Reduced Quadrilateral Element (S4R)	Triangular Element (S3)	QLLL Element
$A_{11},\;\left(\mathrm{kPa}\cdot m\right)$	2219	2218	2225	2128
$A_{22},\;\left(\mathrm{kPa}\cdot m\right)$	1694	1694	1694	1677
$A_{12},\;\left(\mathrm{kPa}\cdot m\right)$	411.8	411.5	413.4	378.9
$A_{33},\;\left(\mathrm{kPa}\cdot m\right)$	659.3	659.3	659.6	659.7
$D_{11},\;\left(\mathrm{Pa}\cdot m^{3}\right)$	6.521	6.517	6.535	6.443
$D_{22},\;\left(\mathrm{Pa}\cdot m^{3}\right)$	4.071	4.066	4.091	4.035
$D_{12},\;\left(\mathrm{Pa}\cdot m^{3}\right)$	1.149	1.148	1.152	1.135
$D_{33},\;\left(\mathrm{Pa}\cdot m^{3}\right)$	1.729	1.728	1.731	1.716
$A_{44}$,$\left(\mathrm{Pa}\cdot m\right)$	140.5	139.8	143.8	71.1
$A_{55}$,$\;\left(\mathrm{Pa}\cdot m\right)$	132.6	132.4	135.6	102.4
nodes/element	969/896	969/896	969/1792	969/896

**Table 4:** The stiffnesses of the representative shell element computed for different number of segments for one fluting period–sine geometry.

Stiffness	Unsymmetric 8 Segments	Unsymmetric 16 Segments	Unsymmetric 32 Segments	Symmetric 8 Segments	Symmetric 16 Segments	Symmetric 32 Segments
$A_{11},\;\left(\mathrm{kPa}\cdot m\right)$	2128	2108	2106	2126	2114	2107
$A_{22},\;\left(\mathrm{kPa}\cdot m\right)$	1677	1681	1682	1678	1681	1682
$A_{12},\;\left(\mathrm{kPa}\cdot m\right)$	378.9	373.7	373.4	380.4	375.9	373.7
$A_{33},\;\left(\mathrm{kPa}\cdot m\right)$	659.7	658.7	658.3	659.6	658.4	658.1
$D_{11},\;\left(\mathrm{Pa}\cdot m^{3}\right)$	6.443	6.433	6.432	6.445	6.435	6.429
$D_{22},\;\left(\mathrm{Pa}\cdot m^{3}\right)$	4.035	4.087	4.101	4.033	4.086	4.099
$D_{12},\;\left(\mathrm{Pa}\cdot m^{3}\right)$	1.135	1.130	1.130	1.137	1.131	1.129
$D_{33},\;\left(\mathrm{Pa}\cdot m^{3}\right)$	1.715	1.728	1.732	1.682	1.694	1.698
$A_{44}$,$\left(\mathrm{Pa}\cdot m\right)$	71.1	48.0	43.1	75.0	49.0	42.5
$A_{55}$,$\;\left(\mathrm{Pa}\cdot m\right)$	102.4	104.4	104.7	113.4	114.4	114.6

**Table 5:** The stiffnesses of the representative shell element computed for different numbers of periods for unsymmetric and symmetric sine geometry.

Stiffness	Unsymmetric 1 Period	Unsymmetric 2 Periods	Unsymmetric 3 Periods	Symmetric 1 Period	Symmetric 2 Periods	Symmetric 3 Periods
$A_{11},\;\left(\mathrm{kPa}\cdot m\right)$	2108	2106	2106	2114	2110	2108
$A_{22},\;\left(\mathrm{kPa}\cdot m\right)$	1681	1680	1680	1681	1681	1681
$A_{12},\;\left(\mathrm{kPa}\cdot m\right)$	373.7	373.4	373.3	375.9	374.5	374.0
$A_{33},\;\left(\mathrm{kPa}\cdot m\right)$	658.7	658.5	658.4	658.4	658.4	658.4
$D_{11},\;\left(\mathrm{Pa}\cdot m^{3}\right)$	6.433	6.445	6.458	6.435	6.428	6.426
$D_{22},\;\left(\mathrm{Pa}\cdot m^{3}\right)$	4.087	4.085	4.085	4.086	4.085	4.084
$D_{12},\;\left(\mathrm{Pa}\cdot m^{3}\right)$	1.130	1.129	1.129	1.131	1.129	1.128
$D_{33},\;\left(\mathrm{Pa}\cdot m^{3}\right)$	1.728	1.713	1.710	1.694	1.694	1.694
$A_{44}$,$\left(\mathrm{Pa}\cdot m\right)$	48.0	45.9	45.1	49.0	46.4	45.4
$A_{55}$,$\;\left(\mathrm{Pa}\cdot m\right)$	104.4	102.8	102.3	114.4	107.8	105.6

## Data Availability

The data presented in this study are available on request from the corresponding author.
